# Accuracy of computer-assisted vertical cup-to-disk ratio grading for glaucoma screening

**DOI:** 10.1371/journal.pone.0220362

**Published:** 2019-08-08

**Authors:** Blake M. Snyder, Sang Min Nam, Preeyanuch Khunsongkiet, Sakarin Ausayakhun, Thidarat Leeungurasatien, Maxwell R. Leiter, Artem Sevastopolsky, Ashlin S. Joye, Elyse J. Berlinberg, Yingna Liu, David A. Ramirez, Caitlin A. Moe, Somsanguan Ausayakhun, Robert L. Stamper, Jeremy D. Keenan

**Affiliations:** 1 School of Medicine, University of Colorado Denver, Aurora, Colorado, United States of America; 2 Francis I. Proctor Foundation, University of California San Francisco, San Francisco, CA, United States of America; 3 Department of Ophthalmology, University of California, San Francisco, San Francisco, CA, United States of America; 4 Department of Ophthalmology, CHA Bundang Medical Center, CHA University, Seongnam, Republic of Korea; 5 Department of Ophthalmology, Faculty of Medicine, Chiang Mai University, Chiang Mai, Thailand; 6 Youth Laboratories LLC, Moscow, Russia; 7 Skolkovo Institute of Science and Technology, Moscow, Russia; University of Florida, UNITED STATES

## Abstract

**Purpose:**

Glaucoma screening can be performed by assessing the vertical-cup-to-disk ratio (VCDR) of the optic nerve head from fundus photography, but VCDR grading is inherently subjective. This study investigated whether computer software could improve the accuracy and repeatability of VCDR assessment.

**Methods:**

In this cross-sectional diagnostic accuracy study, 5 ophthalmologists independently assessed the VCDR from a set of 200 optic disk images, with the median grade used as the reference standard for subsequent analyses. Eight non-ophthalmologists graded each image by two different methods: by visual inspection and with assistance from a custom-made publicly available software program. Agreement with the reference standard grade was assessed for each method by calculating the intraclass correlation coefficient (ICC), and the sensitivity and specificity determined relative to a median ophthalmologist grade of ≥0.7.

**Results:**

VCDR grades ranged from 0.1 to 0.9 for visual assessment and from 0.1 to 1.0 for software-assisted grading, with a median grade of 0.4 for each. Agreement between each of the 8 graders and the reference standard was higher for visual inspection (median ICC 0.65, interquartile range 0.57 to 0.82) than for software-assisted grading (median ICC 0.59, IQR 0.44 to 0.71); P = 0.02, Wilcoxon signed-rank test). Visual inspection and software assistance had similar sensitivity and specificity for detecting glaucomatous cupping.

**Conclusion:**

The computer software used in this study did not improve the reproducibility or validity of VCDR grading from fundus photographs compared with simple visual inspection. More clinical experience was correlated with higher agreement with the ophthalmologist VCDR reference standard.

## Introduction

Advances in portable, non-invasive imaging and diagnostic technologies have made tele-ophthalmology a potential strategy to screen for eye diseases such as diabetic retinopathy and glaucoma.[1] Non-ophthalmologist technicians perform fundus photography in some tele-ophthalmology programs, but interpretation of images remains a challenge.[2] Photographs are typically transferred via a store-and-forward method to expert reading centers, where they are interpreted at a later time.[3, 4] Although theoretically possible, real time interpretation at a reading center remains difficult since it requires a fast reliable internet connection and sufficient grading staff. A simpler strategy would use a test that provides a result at the time of screening without the need for an expert grader. While automated algorithms are increasingly filling this need for diabetic retinopathy screening[5–7], few algorithms have been developed to classify images in terms of glaucomatous optic neuropathy. [8]

Software-assisted grading of fundus photographs has the potential to improve tele-ophthalmology programs by allowing non-ophthalmologists to more easily identify clinically relevant glaucomatous disease metrics. We make available here a simple ImageJ plugin to assist with grading the vertical cup-to-disk ratio (VCDR) of the optic nerve head (ONH) from routine fundus photographs. Although glaucoma diagnosis is complex and not based on any single metric, the VCDR is one of the most important clinical features used when making a diagnosis of glaucoma and likewise one of the most important predictors for glaucoma progression.^7^ In the context of telemedicine programs, a high VCDR would greatly raise suspicion of glaucoma and trigger referral to an ophthalmologist. However, VCDR assessment is inherently subjective and inter-grader variability is high, even among ophthalmologists.[9–14] We test in the present study whether our software made assessment of the VCDR more reliable between graders. If so, this would support the idea of non-expert graders performing VCDR assessment at the point of care, without the need for a reading center.

## Materials and methods

### Study design and population

This was a cross-sectional diagnostic accuracy study using publicly available data. We chose an arbitrary set of 200 unique fully anonymized images from two open-access retinal image databases (i.e., 162 images from the RIM-ONE v1 database and 38 images from the Hunter database) that were considered to be of adequate quality (i.e., good focus, acceptable media opacity).[15, 16] This study is not human subjects research and did not require ethical approval. We followed GRRAS reporting guidelines for studies of reliability and agreement.

### Reference standard

A group of 5 board-certified ophthalmologists independently assessed the VCDR of each image to the nearest tenth without the computer software. All ophthalmologists were in clinical practice at universities, with 4, 10, 11, 11, and 12 years of post-residency clinical experience. The median of these 5 estimates was used as the reference standard grade for each image.

### Diagnostic test

A group of 8 non-ophthalmologists (4 medical students/interns planning on post-graduate ophthalmology training and 4 individuals without clinical experience) and two ophthalmologists graded each image both visually and with software assistance. All graders received a short training module and a reference sheet depicting VCDR grades from 0.1 to 0.9 in 0.1 increments that was to be used during grading. All medical student and intern graders had familiarity with VCDR grading during their clinical ophthalmology rotations. The other four graders had no prior experience with VCDR grading nor with clinical care in general. Specifically, the 200 images were duplicated and labelled with 400 unique identification tags. Graders were presented with 4 sets of 100 randomly ordered images; each set was graded alternately by visual inspection or with computer assistance. Graders were masked to the results of other graders, and by nature of the design, did not grade the same image visually and with software-assistance consecutively. The optic nerve cup and disk border were determined using color, contour, and vasculature cues. Grades performed visually were reported to the nearest tenth and grades calculated with software-assistance were rounded up to the nearest tenth. No other information was recorded or used to determine VCDR measurements (e.g., notching, hemorrhage). Graders were asked to document the time spent grading with each method. To determine intra-grader reliability, each grader repeated grading on a renamed random 20% of photographs (4 sets of 20 photos) in a masked fashion. All graders were given the same training module before grading study photos.

### Software-assisted grading plugin

When designing this study, we were unaware of any open-access computer software for VCDR grading, which we found challenging for telemedicine programs in resource-limited settings. Therefore, we developed a custom-made ImageJ plugin for this study (S1 File). The plugin utilizes the ImageJ application program interface with the Region of Interest (ROI) Manager. The grader uses the native ImageJ ellipse tool to draw two ellipses: one for the outline of the disk and the other for the outline of the cup. The program then calculates the length of the longest vertical meridian of each ellipse and reports the VCDR as the ratio of these two lengths. The protocol for use is described in greater detail in S2 File.

### Automated algorithm

Each of the images was also processed with a modification of a previously described software program that provides an estimate of VCDR using a convolutional neural network to segment the optic disk and cup.[8] The optic disc was segmented by a neural network trained on either the RIM-ONE v3 photoset (for the 162 RIM-ONE v1 images) or the DRIONS-DB photoset (for the 38 Hunter images). All images underwent optic cup segmentation by a neural network trained on the RIM-ONE v3 database. The training procedure has been described in detail elsewhere.[8] For optic disc processing, the resolution of the input images was set to 256 x 256; for the optic cup, a region of interest containing the optic disc was cropped from the 512 x 512 image and resized to 128 x 128 with bilinear interpolation. Contrast-limited adaptive histogram equalization was applied in a preprocessing stage to adjust the brightness of images. The modified U-Net network was then applied, returning a segmentation mask for the structure for which the algorithm was trained (i.e., optic disc or optic cup). The network was trained with real-valued dice approximation loss optimized by stochastic gradient descent, with a momentum of 0.95, batch size of 1, and learning rate set to 0.001 for optic disc segmentation and 0.0003 for optic cup segmentation. Data augmentation was used to enlarge the training set by artificial examples; images were subject to random rotations, zooms, shifts and flips.

### Statistical analysis

The repeatability (i.e., intra-grader agreement) of each method was estimated separately for each of the 8 non-ophthalmologists with an absolute-agreement intraclass correlation coefficient (ICC) for a single rater.[17] The same ICC method was used to estimate the reproducibility (i.e., inter-grader agreement) between graders.[17] The validity of each method was estimated in two ways. First, the agreement between each non-ophthalmologist and the reference standard was computed with the consistency-agreement ICC for a single rater. Second, the non-ophthalmologist VCDRs was dichotomized using three thresholds (i.e., ≥0.6, ≥0.7, and ≥0.8), and sensitivity and specificity of each threshold calculated relative to a reference standard grade of ≥0.7. We repeated the analysis for alternate reference standard thresholds as a sensitivity analysis. A paired Wilcoxon signed-rank test was used to compare ICC estimates between the two grading techniques, and an unpaired Wilcoxon rank-sum test was used to compare ICCs between the graders with and without clinical experience. The possibility for systematic differences between the two methods was assessed by subtracting the inspection-only VCDR from the software-assisted VCDR estimate for each of the 200 photographs. The distribution of these difference scores was visualized as box plots stratified by the reference standard VCDR, and statistical significance was assessed from a mixed effects linear regression that modeled the difference scores across the reference standard VCDRs and included grader as a random effect. The joint sensitivity and specificity outcomes were compared between experienced and inexperienced graders and between the two VCDR estimation methods using a MANOVA adjusted for grader. Statistical analysis was performed in R (R Foundation for Statistical Computing, Vienna, Austria).[18]

## Results

**Fig 1:**
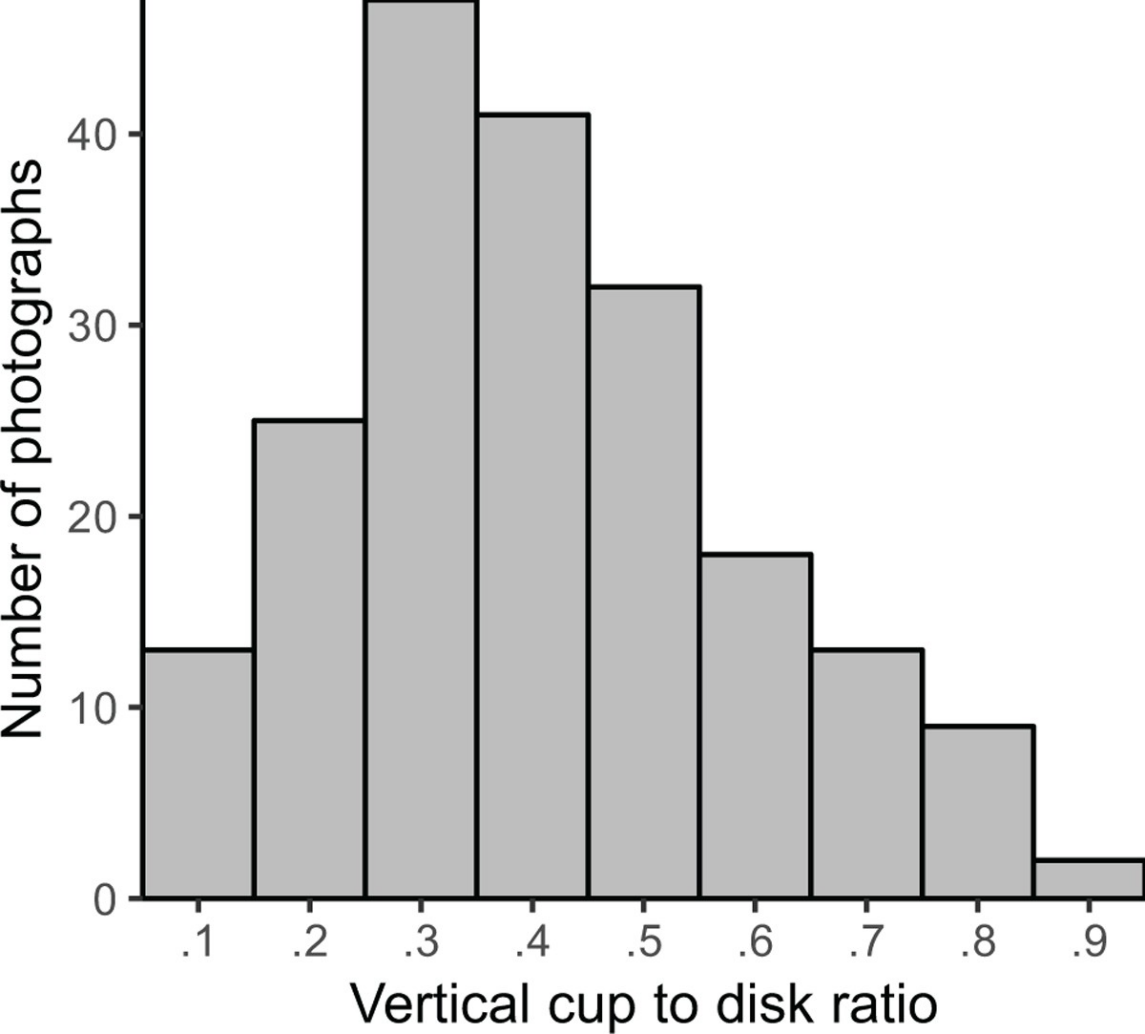
Distribution of reference standard vertical cup-to-disk ratio (VCDR) grades. The histogram shows the distribution of the median VCDR grade given by a group of 5 ophthalmologists on a set of 200 optic disk photographs.

The 5 ophthalmologists assigned VCDRs ranging from 0.1–0.9 with a median grade of 0.4 within the dataset. Inter-ophthalmologist reproducibility was acceptable, with an ICC of 0.79 (95% CI 0.75 to 0.83). In a sensitivity analysis, two of the ophthalmologists also performed VCDR grading with the software program; agreement between these two ophthalmologists was higher for visual inspection (ICC 0.73, 95% CI 0.57 to 0.82) than for software-assisted grading (ICC 0.44, 95% CI 0.04 to 0.67). The median of the 5 VCDR grades was used as the reference standard for the remaining analyses; the distribution of these reference standard grades is shown in Fig 1.

The 8 non-ophthalmologists assigned grades ranging from 0.1 to 0.9 with visual inspection (median 0.4, interquartile range [IQR] 0.3 to 0.5) and from 0.1 to 1 with software assistance (median 0.4, IQR 0.4 to 0.5). Median grading time differed significantly between the two methods, taking on average 31 seconds per image (95%CI 17 to 46 seconds) for visual inspection and 57 seconds per image (95%CI 32 to 82 seconds) for computer-assisted grading (P = 0.007, Wilcoxon signed-rank test).

Intra-grader repeatability is shown for each grader and method in Table 1. Graders with clinical experience tended to have higher repeatability than those without experience, both for visual and software-assisted grading (P = 0.03 and P = 0.03 respectively, Wilcoxon rank-sum test). Repeatability with the visual inspection method was not statistically significantly different from that of the computer-assisted method (P = 0.69, Wilcoxon signed-rank test). When the 8 graders were compared with each other, inter-grader reproducibility was higher for visual inspection (ICC 0.51, 95%CI 0.45 to 0.57) than software-assisted grading (ICC 0.44, 95%CI 0.38 to 0.51).

**Table 1:** Repeatability and validity of visual inspection and software-assisted determination of the vertical cup-to-disk ratio (VCDR) by non-ophthalmologists.

	Intra-grader repeatability*		Agreement to reference standard^†^	
Grader	Visual	Software-Assisted	Visual	Software-Assisted
No clinical training				
1	0.79 (0.64–0.88)	0.37 (0.07–0.61)	0.35 (0.22–0.47)	0.23 (0.09–0.35)
2	0.44 (0.16–0.66)	0.45 (0.17–0.67)	0.59 (0.50–0.68)	0.49 (0.38–0.59)
3	0.54 (0.28–0.72)	0.72 (0.53–0.84)	0.58 (0.48–0.67)	0.43 (0.31–0.54)
4	0.53 (0.27–0.72)	0.56 (0.30–0.74)	0.57 (0.46–0.65)	0.55 (0.45–0.64)
Clinical training				
5	0.90 (0.84–0.95)	0.84 (0.72–0.91)	0.82 (0.77–0.86)	0.73 (0.66–0.79)
6	0.88 (0.79–0.94)	0.89 (0.80–0.94)	0.80 (0.75–0.85)	0.63 (0.54–0.71)
7	0.87 (0.77–0.93)	0.85 (0.79–0.94)	0.72 (0.64–0.78)	0.70 (0.62–0.77)
8	0.83 (0.70–0.90)	0.66 (0.44–0.80)	0.82 (0.77–0.86)	0.71 (0.63–0.77)

* Absolute-agreement ICC for a set of 25 pairs of duplicate images

^**†**^ Consistency-agreement ICC comparing the non-ophthalmologist grader with the reference standard grade on a set of 200 unique images.

Values represent intraclass correlation coefficient (ICC) estimates for each grading method with 95% confidence intervals in parentheses, stratified by grader.

**Fig 2:**
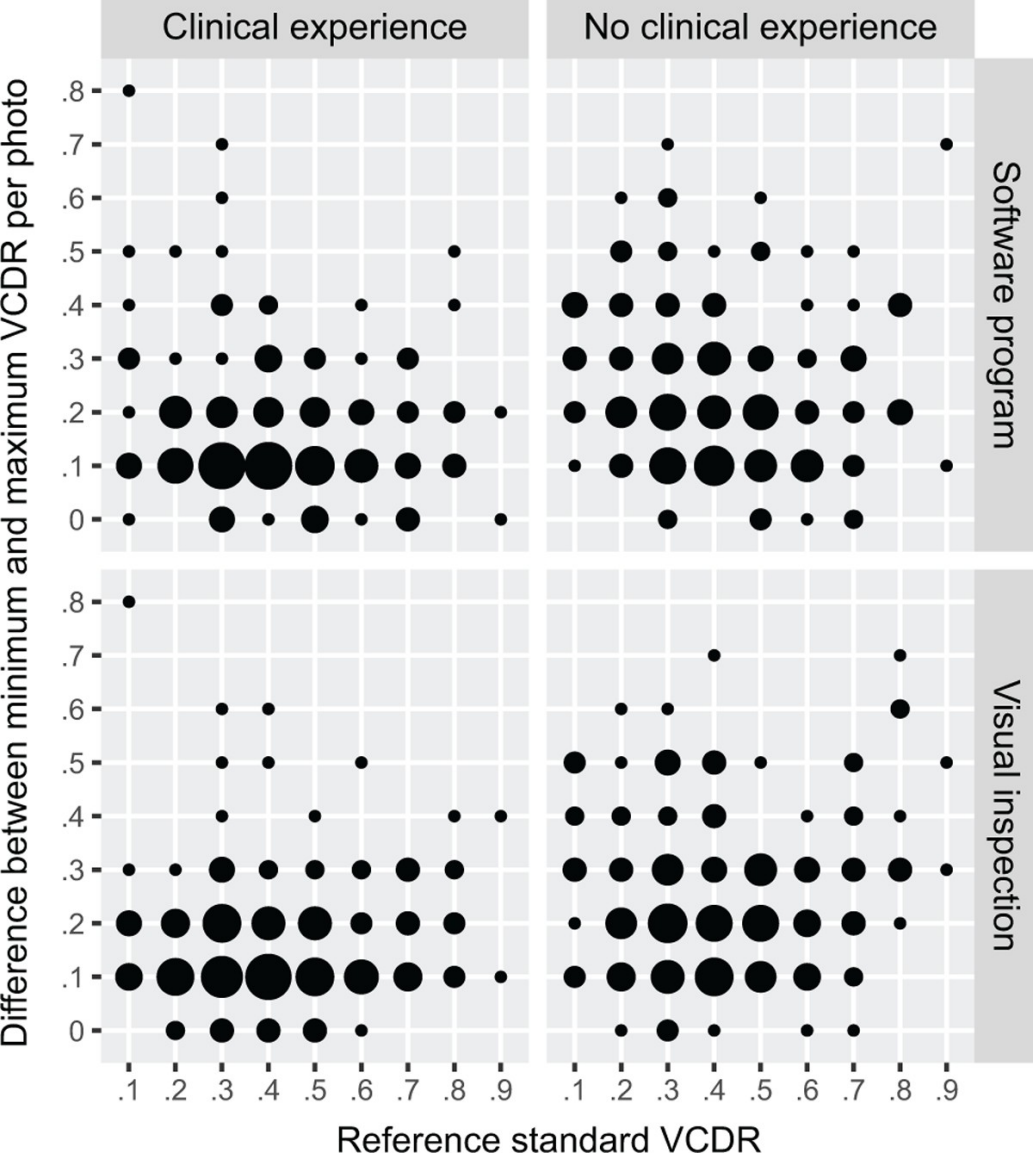
Range of VCDR grades given by non-ophthalmologist graders. The difference between the minimum and maximum VCDR grades given by the non-ophthalmologist graders is plotted against the reference standard VDCR grade. Plots are shown separately for the four graders with clinical experience (left panels) and four graders without clinical experience (right panels), and also for the software-assisted (top panels) and visual inspection (bottom panels) grading techniques. The area of the dots is proportional to the number of observations.

As shown in Table 1, agreement between each of the 8 graders and the reference standard was higher for visual inspection (median ICC 0.65, IQR 0.57 to 0.82) than for computer-assisted grading (median ICC 0.59, IQR 0.44 to 0.71); P = 0.02, Wilcoxon signed-rank test. The 4 graders with clinical experience had better agreement with the reference standard than the 4 graders without clinical experience (P = 0.03 for visual inspection and P = 0.03 for software-assisted grading, Wilcoxon rank-sum test). Moreover, the variability in grades for the four graders without clinical experience tended to be greater than that of the four graders with clinical experience (Fig 2). The automated algorithm produced identical results when run on the set of duplicate images, but had less agreement with the reference standard than the other two grading methods (ICC 0.36, 95%CI 0.23 to 0.47).

**Fig 3:**
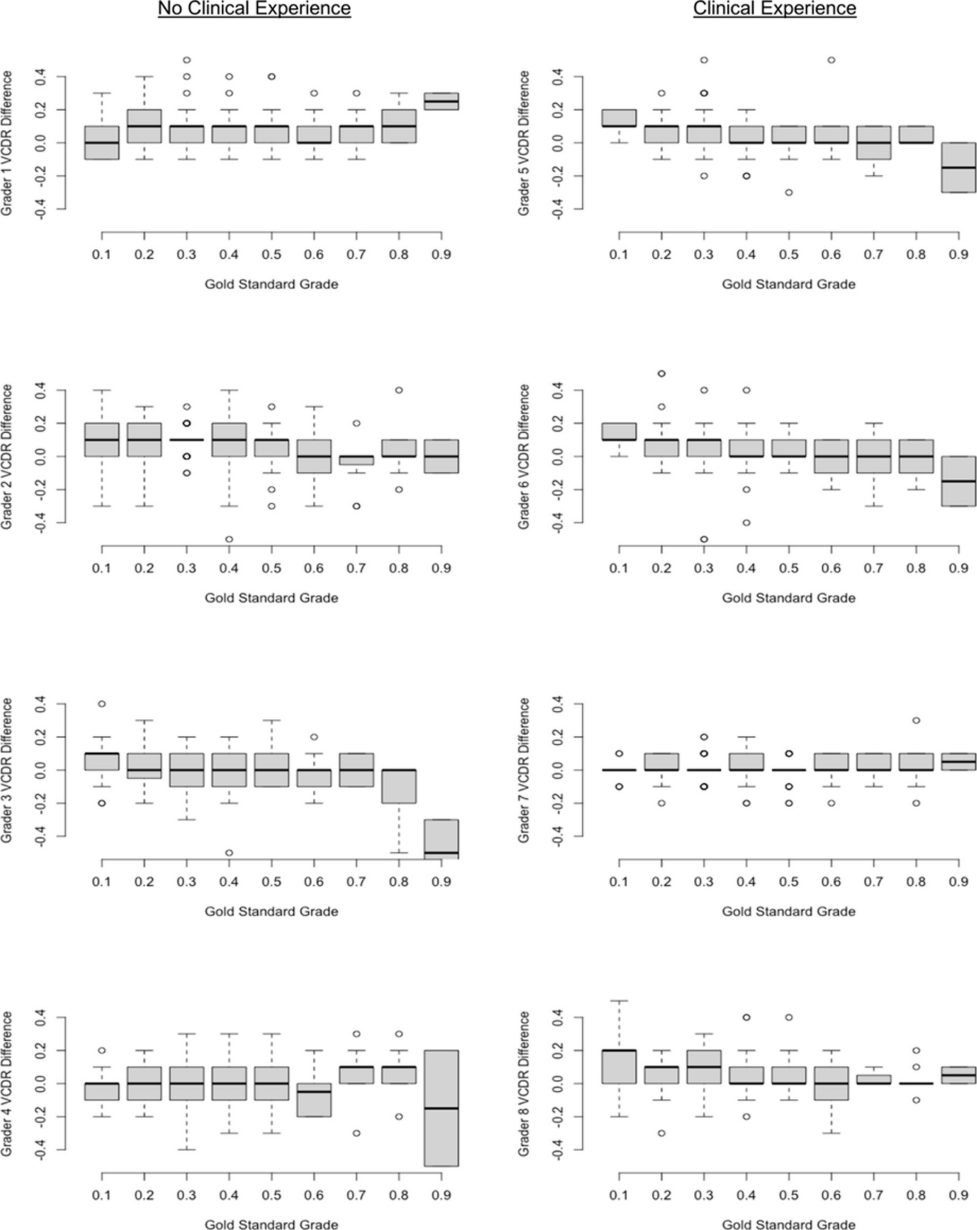
Difference of visual to software-assisted grading across different VCDR Values. Positive numbers mean that the program overestimated visual inspection, whereas negative numbers mean that the program underestimated visual inspection.

The difference between the visual and software-assisted VCDR grade was calculated for each non-ophthalmologist in order to assess for systematic differences between the two grading methods. On average, software-assisted grading overestimated visual inspection (mean among the 8 graders: 0.05 units higher, 95%CI 0.02 to 0.08). We plotted the difference between the two methods as a series of box plots stratified by the reference standard grade in order to determine whether the computer software performed differently depending on the severity of optic nerve cupping (Fig 3). In general, the computer program tended to provide an overestimate at lower VCDRs and an underestimate at higher VCDRs (P<0.001, mixed effects linear regression).

**Fig 4:**
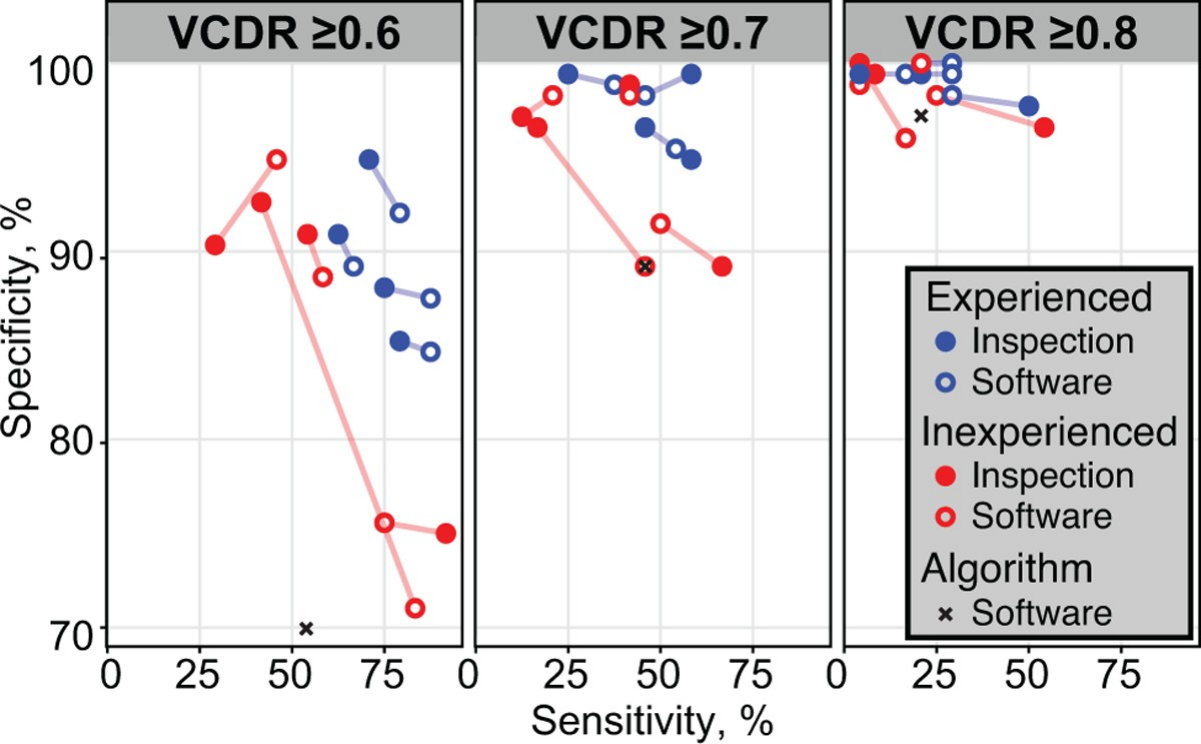
Diagnostic accuracy for classification of optic disk cupping. Four individuals with and four individuals without clinical experience graded a set of fundus photographs for vertical cup-to-disk ratio (VCDR) using two different methods: visual inspection and with software assistance. The sensitivity and specificity for each grader was calculated relative to a reference standard VCDR of ≥0.7, assessed as the median of five ophthalmologists grades. A pair of points is shown for each grader, with the filled circle representing visual inspection, the empty circle representing software-assisted grading, and a line joining the pair. An automated algorithm was used to classify VCDR. The three graphs show the sensitivity and specificity for three different thresholds of cupping; note that a perfectly sensitive and specific test would be located in the upper right-hand corner of each plot.

The non-ophthalmologist VCDR grades were then analyzed as a diagnostic test for glaucomatous cupping, with the reference standard defined as a median ophthalmologist VCDR of ≥0.7. Sensitivity and specificity were calculated for three different diagnostic test thresholds (i.e., non-ophthalmologist VCDRs of ≥0.6, ≥0.7, and ≥0.8) separately for each of the 8 graders and for each of the two methods. Sensitivities and specificities are depicted in a series of scatter plots in which the diagnostic accuracy estimates can be classified by test threshold, grading method, clinical experience, and grader (Fig 4). Sensitivity was generally low, although tended to be higher for experienced graders (blue circles) compared with inexperienced graders (red circles); MANOVA P = 0.003, P = 0.001, and P = 0.07 for VCDR thresholds of ≥0.6, ≥0.7, and ≥0.8, respectively, but did not differ by grading method (i.e., filled circles vs. empty circles; P = 0.34, P = 0.75, and P = 0.80, respectively). Results did not differ when different reference standard thresholds for glaucomatous cupping were used (S1 Fig). Conclusions did not change in a sensitivity analysis of the RIM-ONE images that assumed the reference standard classification provided by the original researchers (N = 162 total images, 38 of which were classified as glaucoma; S1 Table).

## Discussion

Accurate measurement of ONH morphology, and in particular VCDR, is essential for diagnosis and management of glaucoma. Software has the potential to make grading more objective and reproducible, especially for non-ophthalmologist graders as might be employed by a telemedicine program. Given the lack of open-access software platforms, we designed a computer-assisted software program to help non-ophthalmologists grade the VCDR. Contrary to our hypothesis, we found that a simple ImageJ plugin was not able to improve the reproducibility or validity of VCDR measurements. In fact, computer-assisted grading performed worse than simple visual inspection, and took considerably longer—likely because it required using the software to manually draw two ellipses per photograph. Use of the software program resulted in systematic underestimation of the VCDR in cases of more severe cupping. Medical students agreed with the reference standard to a greater degree than non-clinical graders, though even medical students were more reliable when using simple visual inspection as opposed to the software. Sensitivity and specificity were not significantly different between the two methods.

The graders in this study agreed more with the reference standard VCDR when using visual inspection than when using computer-assisted grading. We found visual inspection to be superior even though the graders completed fairly minimal training: just a 30-minute one-on-one training session with standardized materials. More intensive training would have been preferred though this was challenging given the volunteer graders’ other commitments. It is possible that further training could have lead to higher accuracy and more agreement across graders, especially given other studies that have shown non-ophthalmologist graders to have higher inter-grader reliability with more extensive training.[19] Despite this, our findings suggest non-ophthalmologist graders might be trained to accurately estimate VCDR using simple visual inspection, although it is important to note that the non-experts with no clinical background did substantially worse than those with clinical experience and interest in ophthalmology, and in some cases, the non-clinical graders demonstrated unacceptable agreement with the reference standard. Indeed, these results provide evidence of the high variability in VCDR grading, especially when done by those with little clinical experience. Nonetheless, it is reasonable to postulate that with a more intensive training experience, non-experts could provide valid real-time VCDR assessment at the point of screening.

The results of the present study could be specific to the particular software we used. We performed cup and disk segmentation with the simple ellipse tool in ImageJ and calculated the VCDR as the proportion of the longest vertical axis of each ellipse. A previous study reported better reproducibility with a software program using a polynomial curve-fitting algorithm, though this study reported results only for two eye care professionals.[20] It would be worthwhile to test the validity and reproducibility of other software programs when used by non-ophthalmologists, though we are not aware of any other programs that are publicly available.

The automated algorithm tested in this study was not better than human grading in terms of agreement or validity relative to an ophthalmologist reference standard. Yet, given the rapid development of artificial intelligence technologies, it is feasible to consider that a system will soon be capable of producing accurate real-time optic disk and cup segmentation, including clinically relevant metrics such as VCDR. Further development and validation of automated algorithms would be beneficial for an eye disease screening program, as an algorithm could theoretically screen for several diseases simultaneously, negating the need for a non-expert to grade fundus photographs at all.

There were several strengths of this study. We used a median grade of 5 ophthalmologists to create a more accurate reference standard, and we compared two grading techniques on a relatively large number of non-expert graders. This design, plus the broad range of VCDRs included in the study, should increase its generalizability to tele-ophthalmology programs. There were also some limitations. The study was performed on a relatively small set of photographs and we did not have access to a corresponding VCDR grade estimated from in-person ophthalmologist exam. Other diagnostic features of glaucomatous disease, such as disk hemorrhage or notching, were not captured, nor were other optic disk metrics such as the horizontal cup to disk ratio. Relatively few images had very high VCDRs, limiting the precision of estimates of sensitivity. Graders assessed only monoscopic images. Stereoscopic fundus photographs may have provided higher reliability metrics, although previous studies have suggested that there is not a significant difference between monoscopic and stereoscopic images.[20–23]

Computer-assisted and automated systems for VCDR grading are enticing as in theory they could limit variability and subjectivity of current grading methods and provide real-time results during photographic screening. In this study, neither our simple ImageJ plugin nor an automated algorithm were superior to visual inspection in terms of agreement with a reference standard VCDR. Recently, automated systems have been developed to compute VCDRs by using fundus photo information, including pixel color variation, border variation, and blood vessel bend.[24–26] Convolutional neural networks utilizing ophthalmologist grades on image datasets have also shown promise.[27–29] However, to our knowledge, none of these automated models are open source platforms and may be cost prohibitive in resource-limited settings. Until such software becomes available, our results suggest that remote screening programs utilizing retinal photography could employ visual inspection at the point of care to provide VCDR estimates, but this would require a training program more intensive than the one used in this study.

## Supporting information

S1 File Custom-made ImageJ plugin for computer-assisted grading of vertical cup-to-disk ratio. (ZIP)

S2 File User manual for a custom-made ImageJ plugin for computer-assisted grading of vertical cup-to-disk ratio. (PDF)

S3 File Dataset. (CSV)

S4 File Data dictionary for dataset. (CSV)

S1 Fig Diagnostic accuracy for classification of optic disk cupping relative to different reference standards. Four individuals with and four individuals without clinical experience graded a set of fundus photographs for vertical cup-to-disk ratio (VCDR) using three different methods: visual inspection, with software assistance, and by automated alogrithm. The sensitivity and specificity for each grader was calculated relative to three reference standard VCDRs (i.e., ≥0.6, ≥0.7, and ≥0.8) assessed as the median of five ophthalmologists grades. A pair of points is shown for each grader, with the filled circle representing visual inspection, the empty circle representing software-assisted grading, and a line joining the pair. The three graphs show the sensitivity and specificity for three different thresholds of cupping; note that a perfectly sensitive and specific test would be located in the upper right-hand corner of each plot.
(PDF)

S1 Table Receiver operating characteristics (ROC) analysis stratified by grader and grading technique. The analysis was performed for 162 RIM-ONE images in which the reference standard was defined as early (N = 12), moderate (N = 12), or deep (N = 14) glaucoma.
(DOCX)
