# Supplementary material for: Accuracy of computer-assisted vertical cup-to-disk ratio grading for glaucoma screening
Source: PLoS One. 2019 Aug 8;14(8):e0220362. doi: 10.1371/journal.pone.0220362 (PMC6687168; doi:10.1371/journal.pone.0220362)
Supplement: S2 File — (PDF) [file pone.0220362.s002.pdf]

ImageJ Glaucoma Grading Plugin

Installation

1. Download ImageJ
    a. You have two options. You can download...
        i. **Fiji** - an ImageJ2 distribution with a large array of plugins out of the box: https://fiji.sc/#download
        ii. **ImageJ1** - an outdated version of ImageJ, but the plugin will still be compatible: https://imagej.nih.gov/ij/download.html
2. Launch the installer

. For **Fiji**:

. On OS X: drag the `Fiji` application to the `Applications` folder

i. On Windows: Store the `Fiji` application anywhere **except** `Program Files`

a. For **ImageJ1:**

. Double-click on the `.jar` file or right-click and open

3. Download Supplementary File 1 Glaucoma Grading (`Glaucoma_Grading.txt`)
4. Rename the downloaded files suffix to `Glaucoma_Grading`**`.class`**
5. Launch ImageJ / Fiji

. Drag the newly created `Glaucoma_Grading.class` file to the ImageJ toolbar

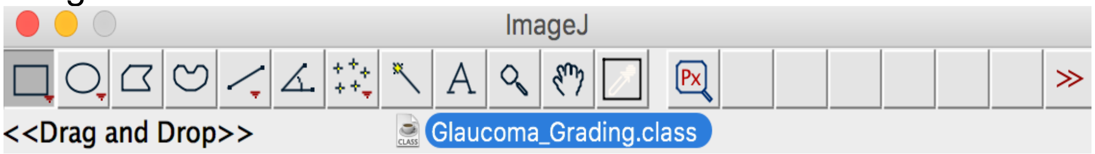

a. Alternatively, go to the `Plugins` menubar option, select `install`, and find and choose `Glaucoma_Grading.class`

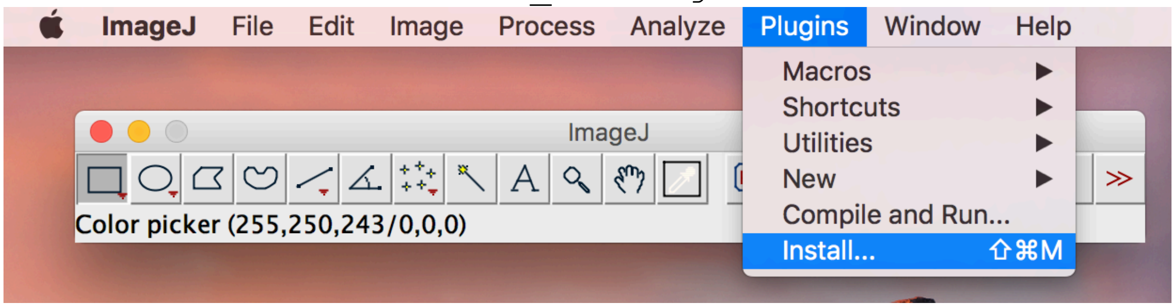

b. It’s recommended you ensure `Glaucoma_Grading.class` exists in the `Plugins/` folder of your ImageJ installation

6. Restart ImageJ / Fiji
7. The plugin is now successfully installed.

Usage

1. Open the first image

a. When grading multiple images, I recommend having them all in one folder for ease of advancing images

2. Go to the menubar option `Plugins` and choose `“Glaucoma Grading”`

. A window titled `“ROI Manager”` should appear

3. Using any selection tool(s), create an ROI (Region of Interest) around either the cup or the disc

. Tips:

i. Hold `shift` when using a selection tool to add to a selection

ii. Hold `alt` when using a selection tool to remove from a selection

iii. Hold `command` and `+` to zoom in, or `command` and `-` to zoom out

iv. Hold `spacebar` to use a mouse tool to navigate the image when zoomed in

v. You can find more tips on the ImageJ wiki: https://imagej.nih.gov/ij/docs/guide/146-10.html

4. Once your selection is finished, press `shift` and `enter` (in sequence, so `shift` THEN `enter`, while still holding down `shift`).

. The ROI should now be present in the `ROI Manager` window. You can click its entry in this window to remove, modify, or copy it.

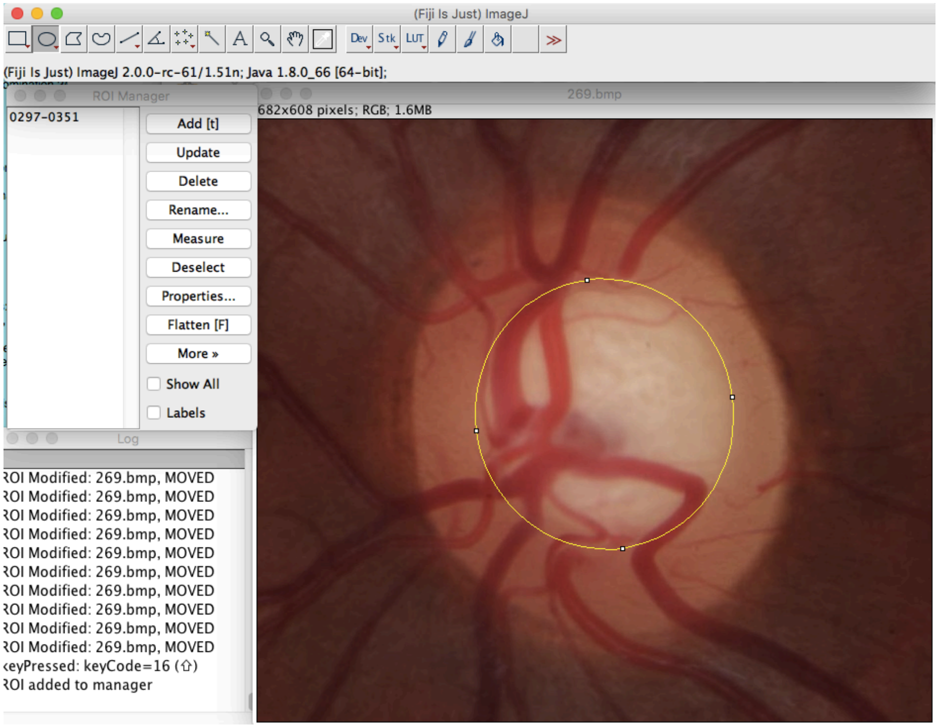

5. Repeat the same selection process for either the cup or the disc -- whichever you did NOT do in steps 3 and 4.

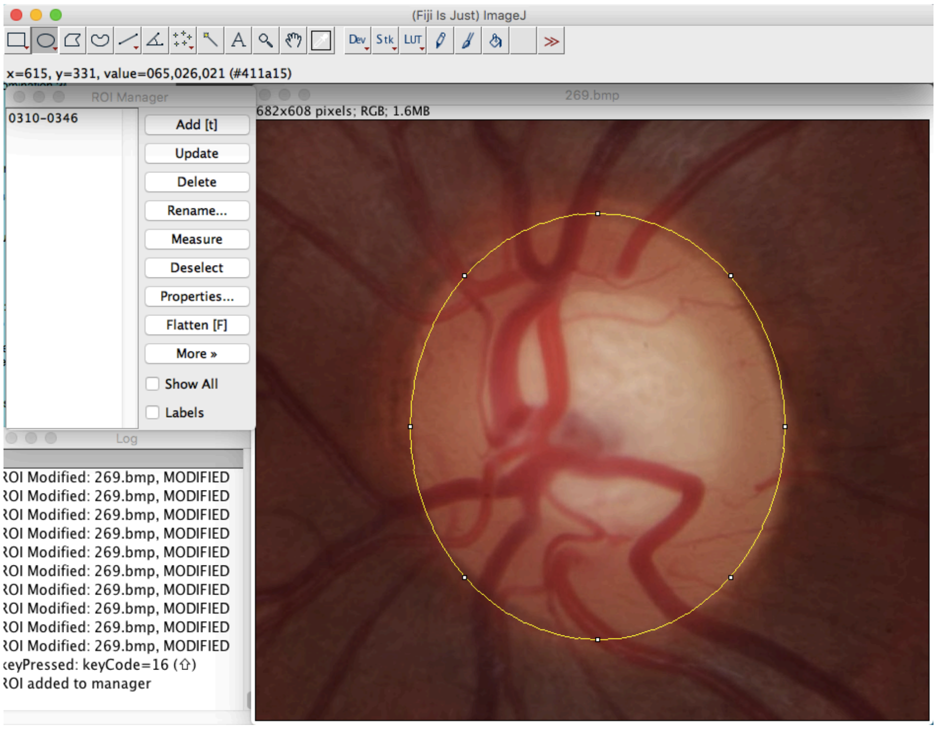

6. Once the second selection is complete, press `shift` and `enter` in sequence again to add the second ROI to the ROI Manager.
7. A "`Results`" window will now appear, containing:

. Vertical Cup-To-Disc

a. Horizontal Cup-To-Disc

b. Square Root of Area Ratio

8. You can now either:

. Open another image

. Using anything in the `File` menu. If the images are in the same folder, you can simply `Open next` to advance images.

a. Save the results

. Press `Command` and `S` or right-click on the `Results` window to save as a CSV
