# Supplementary material for: Accuracy of computer-assisted vertical cup-to-disk ratio grading for glaucoma screening
Source: PLoS One. 2019 Aug 8;14(8):e0220362. doi: 10.1371/journal.pone.0220362 (PMC6687168; doi:10.1371/journal.pone.0220362)
Supplement: S1 Fig — Four individuals with and four individuals without clinical experience graded a set of fundus photographs for vertical cup-to-disk ratio (VCDR) using three different methods: visual inspection, with software assistance, and by automated alogrithm. The sensitivity and specificity for each grader was calculated relative to three reference standard VCDRs (i.e., ≥0.6, ≥0.7, and ≥0.8) assessed as the median of five ophthalmologists grades. A pair of points is shown for each grader, with the filled circle representing visual inspection, the empty circle representing software-assisted grading, and a line joining the pair. The three graphs show the sensitivity and specificity for three different thresholds of cupping; note that a perfectly sensitive and specific test would be located in the upper right-hand corner of each plot. (PDF) [file pone.0220362.s005.pdf]

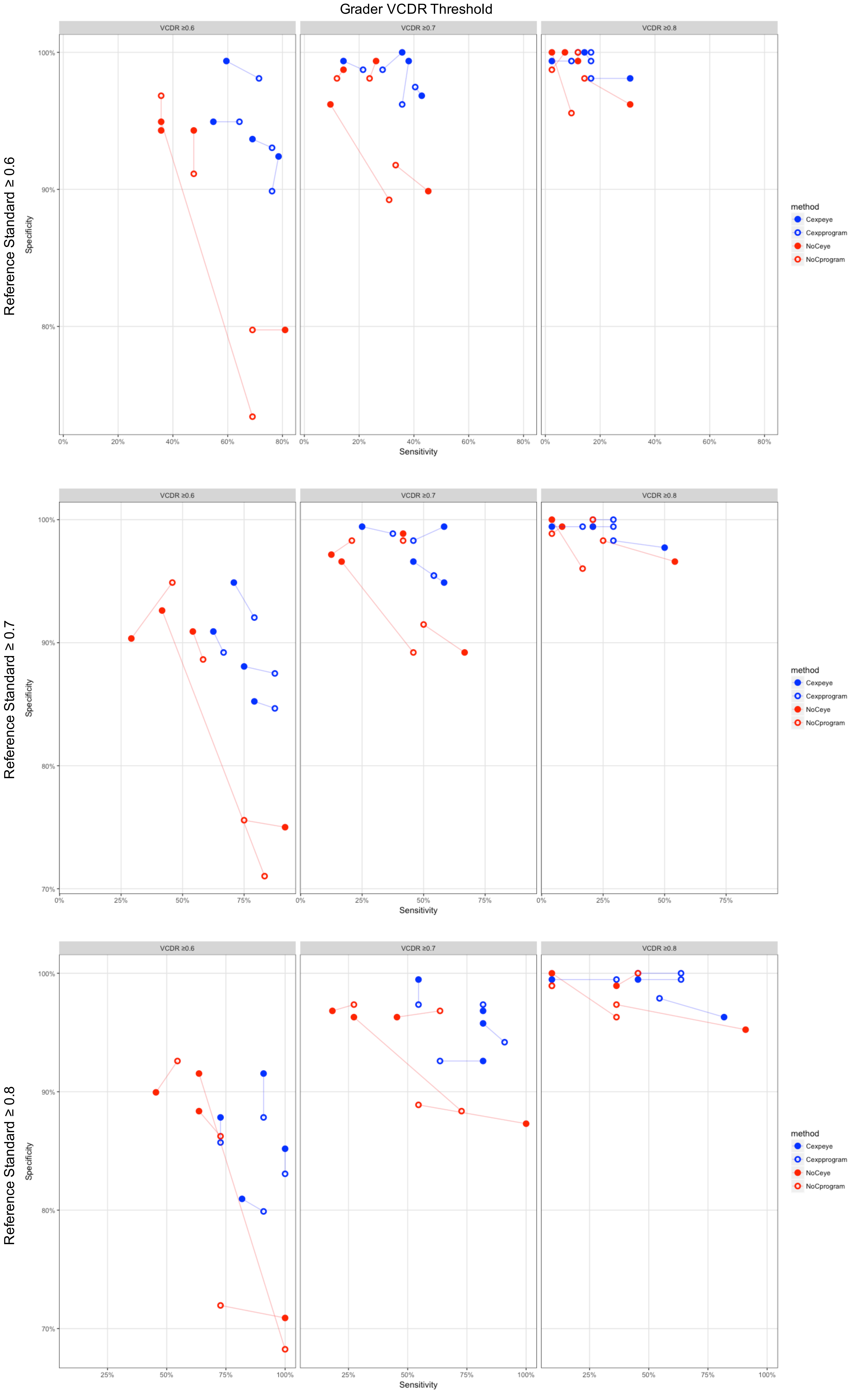

Diagnostic accuracy for classification of optic disk cupping. Four individuals with and four individuals without clinical experience graded a set of fundus photographs for vertical cup-to-disk ratio (VCDR) using two different methods: visual inspection and with software assistance. The sensitivity and specificity for each grader was calculated relative to a reference standard VCDR of ≥0.6, ≥0.7, ≥0.8 assessed as the median of five ophthalmologists grades. A pair of points is shown for each grader, with the filled circle representing visual inspection, the empty circle representing software-assisted grading, and a line joining the pair. The three graphs show the sensitivity and specificity for three different thresholds of cupping; note that a perfectly sensitive and specific test would be located in the upper right-hand corner of each plot.
