# Supplementary material for: Accuracy of computer-assisted vertical cup-to-disk ratio grading for glaucoma screening
Source: PLoS One. 2019 Aug 8;14(8):e0220362. doi: 10.1371/journal.pone.0220362 (PMC6687168; doi:10.1371/journal.pone.0220362)
Supplement: S1 Table — The analysis was performed for 162 RIM-ONE images in which the reference standard was defined as early (N = 12), moderate (N = 12), or deep (N = 14) glaucoma. (DOCX) [file pone.0220362.s006.docx]

| **S6 Table. Receiver operating characteristics (ROC) analysis stratified by grader and grading technique.** The analysis was performed for 162 RIM-ONE images in which the reference standard was defined as early (N=12), moderate (N=12), or deep (N=14) glaucoma. | | |
| --- | --- | --- |
|  | Area under the curve (AUC) | |
| Grader | Visual inspection | Software-Assisted |
| Non-ophthalmologists |  |  |
| No clinical training |  |  |
| 1 | 0.68 | 0.80 |
| 2 | 0.73 | 0.67 |
| 3 | 0.80 | 0.70 |
| 4 | 0.63 | 0.72 |
| Clinical training |  |  |
| 1 | 0.74 | 0.69 |
| 2 | 0.74 | 0.72 |
| 3 | 0.74 | 0.74 |
| 4 | 0.76 | 0.76 |
| Ophthalmologists |  |  |
| 1 | 0.81 | 0.82 |
| 2 | 0.74 | 0.76 |
| 3 | 0.81 | --- |
| 4 | 0.81 | --- |
| 5 | 0.76 | --- |
| *P*=0.06, Kruskal-Wallis test comparing four groups: visual inspection by non-ophthalmologists (N=8), software-assistance by non-ophthalmologists (N=8), visual inspection by ophthalmologists (N=5), software-assistance by ophthalmologists (N=2). | | |
